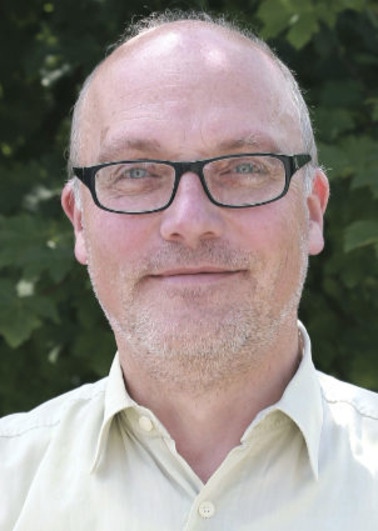# Task‐Specific Janus Materials in Heterogeneous Catalysis

**DOI:** 10.1002/anie.202206403

**Published:** 2022-08-25

**Authors:** Majid Vafaeezadeh, Werner R. Thiel

**Affiliations:** ^1^ Fachbereich Chemie Technische Universität Kaiserslautern Erwin-Schrödinger-Strasse 54 67663 Kaiserslautern Germany

**Keywords:** Green Catalytic Reactions, Heterogeneous Catalysis, Interfacial Catalysis, Janus Catalyst, Pickering Emulsion

## Abstract

Janus materials are anisotropic nano‐ and microarchitectures with two different faces consisting of distinguishable or opposite physicochemical properties. In parallel with the discovery of new methods for the fabrication of these materials, decisive progress has been made in their application, for example, in biological science, catalysis, pharmaceuticals, and, more recently, in battery technology. This Minireview systematically covers recent and significant achievements in the application of task‐specific Janus nanomaterials as heterogeneous catalysts in various types of chemical reactions, including reduction, oxidative desulfurization and dye degradation, asymmetric catalysis, biomass transformation, cascade reactions, oxidation, transition‐metal‐catalyzed cross‐coupling reactions, electro‐ and photocatalytic reactions, as well as gas‐phase reactions. Finally, an outlook on possible future applications is given.

## Graphical abstract

This Minireview summarizes recent advances in the application of nano‐ and microstructured Janus materials, which are a new generation of heterogeneous catalysts for various chemical reactions. Such materials can act as catalysts for reduction, oxidative desulfurization, dye degradation, asymmetric catalysis, biomass transformation, cascade reactions, oxidation, transition‐metal‐catalyzed cross‐coupling reactions, electro‐ and photocatalytic reactions, and gas‐phase reactions.

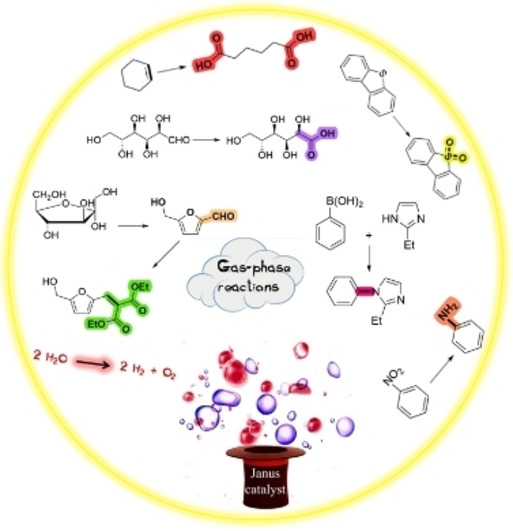

## 1 Introduction

Janus(‐type) materials are particles with at least two different, anisotropically distributed functionalities on the surface, for which remarkable applications have been found in recent years. The term “Janus” originates from the Roman God Janus, who has a head with two faces looking in opposite directions: to the future and to the past. The term “Janus materials” was first used by Casagrande in 1989 to describe spherical glass particles with hydrophobic/hydrophilic hemispheres^[1]^ and became more and more popular after the Nobel Prize Lecture by Pierre‐Gilles de Gennes entitled “Soft Matter”.^[2]^

Since then, research into Janus‐type nano‐ and microarchitectures has attracted attention in diverse fields of material science.^[3]^ Janus particles having different shapes and chemical composition range from silica gels,^[4]^ organic polymers,^[5]^ 2D graphene,^[6]^ up to cellulose,^[7]^ with applications in different fields such as biomedicine,^[8]^ liquid or gas separation and purification,^[9]^ as well as (bio)sensing.^[10]^ Janus particles with oppositely charged hemispheres exhibit large dipole moments, which may be exploited for remote positioning in an electric field, whereas those particles with both electrical and color anisotropy may be used for electronic paper.^[11]^

Various forms of Janus materials have been successfully employed in battery technology including lithium‐ion,[^12a^, ^12b^] lithium‐sulfur,^[12c]^ sodium‐ion,^[12d]^ sodium‐sulfur,^[12e]^ sodium‐oxygen,^[12f]^ and zinc‐air batteries,[^12g^, ^12h^] as well as a nonflammable solid electrolyte for lithium‐metal batteries.^[12i]^ To briefly address the last topic: Meng et al. prepared a composite with a poly(vinyl formal) (PVFM) based Janus membrane, which was coated by multiwalled carbon nanotubes (MWCNTs) on the porous side of a cross‐linked PVFM membrane.^[13]^ The material was suggested as a membrane‐supported gel‐polymer electrolyte (GPE) for lithium‐oxygen batteries. The Janus‐membrane‐supported GPE is responsible for extending and maintaining the stable triple‐phase boundary for the oxygen reduction reaction (ORR) and the oxygen evolution reaction (OER) as well as reducing the interface resistance between the cathode and the electrolyte. The resulting battery showed a remarkable circulation of 150 cycles, a narrow voltage gap, a maximal discharge capacity, as well as an excellent rate performance. As an alternative, Liu et al. fabricated a Janus conductive/insulating microporous membrane for the stabilization of lithium‐sulfur batteries by the deposition of different metal‐organic frameworks (MOFs) on graphene nanosheets.^[14]^ They could show that when a Janus ZIF‐67/graphene nanosheet‐based membrane was used as an ion sieve, a capacity retention of 75.3 % after 1700 cycles was achieved.

As a consequence of their fascinating surface properties, such as tunable polarities and functionalities, Janus‐type materials have found extraordinary attention in heterogeneous catalysis. Although most of the previous reviews on Janus‐type materials have focused on the strategies applied for the fabrication of organic/inorganic Janus‐type nanoarchitectures with distinct surface properties,^[15]^ the application of Janus‐type materials has exponentially grown, in particular during the last decade. The rational design of amphiphilic Janus composites with the co‐existence of hydrophobic and hydrophilic species enables them to act as a heterogeneous surfactant for the stabilization of immiscible water‐in‐oil or oil‐in‐water emulsion systems.^[16]^ Unlike many homogeneous surfactants, which, for example, contain toxic quaternary ammonium cations accompanied by halogenide counter anions, Janus‐type surfactants have a significantly lower negative environmental impact that also benefits from the efficient recovery of the materials by simple filtration. Therefore, such smart Janus‐type materials offer an excellent opportunity to prepare amphiphilic heterogeneous catalysts for performing various chemical transformations in so‐called Pickering emulsion systems. The expression “Pickering emulsion” defines an emulsion that is stabilized by solid particles, which adsorb to the interface between the two phases.^[17]^ Herein, the Janus material acts as a heterogeneous surfactant/phase‐transfer catalyst (PTC), which in addition carries the catalytically active species. Thanks to the usually high specific surface area of such Janus particles, the loading of active catalytic species can be high and uniform, which allows the design and the preparation of high‐performance heterogeneous catalysts. Recently, some reviews on the application of such Janus‐type materials have been published,^[18]^ but they focused on a rather limited number of catalytic applications, since the development of heterogeneous Janus‐type catalysts was still in its early stage.

The term “task‐specific” in the title of this Minireview means that the role of the Janus materials has gone far beyond their initially surfactant‐like or emulsifying properties.^[2]^ This Minireview systematically focuses on the recent and significant advances in (electro‐)chemical catalytic applications of task‐specific heterogeneous Janus nanocatalysts and deals with specific Janus materials used in the concept of a new generation of heterogeneous catalysts. Details on the fabrication methods for Janus materials, which have been previously reviewed, are generally omitted here.

## 2 Catalytic Applications

### 2.1 Reduction

**Figure 1:**
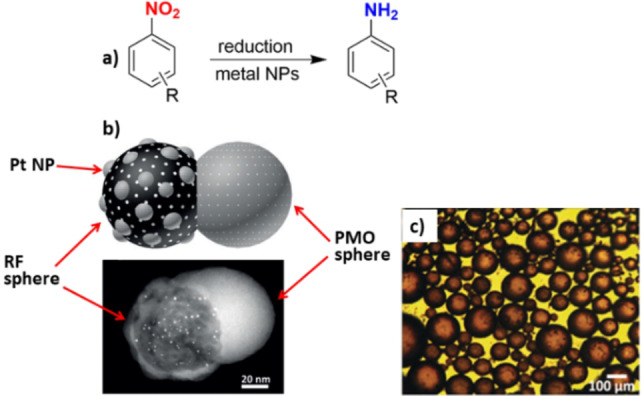
a) Reduction of nitroaromatic compounds with NaBH_4_ in the presence of a metal nanoparticle based Janus catalyst. b) The schematic structure of the dumbbell‐shaped mesoporous carbon‐PMO material decorated with platinum nanoparticles and the TEM image of the catalyst corresponding to the graphical demonstration. c) Optical micrograph of a Pt/C&PMO‐stabilized water/toluene emulsion. Reprinted and adapted from Ref. [27] with permission. © John Wiley and Sons (2017).

Nitroaromatic compounds, and nitrophenol derivatives in particular, are toxic and persistent water pollutants coming from industrial aqueous waste streams. The presence of the nitro group increases the stability of such compounds against chemical degradation, especially against oxidation reactions. In the past, various treatment methods for the degradation of nitroaromatic compounds were developed, which include photocatalytic procedures,^[19]^ Fenton oxidation,^[20]^ and biodegradation.^[21]^ However, most of these processes are energy‐intensive, costly, or inefficient. Janus materials have been widely investigated as supports for metal nanoparticles. Consequently, transition‐metal nanoparticles decorating the surface of Janus‐type materials were found to be efficient catalysts for the reduction of nitroaromatic compounds (Figure 1a).^[22]^ It was found that the reduction of 4‐nitrophenol with NaBH_4_ occurred spontaneously, while a very slow kinetic was observed in the absence of a metal catalyst.^[23]^ A Janus material having an anisotropic surface may act as a complexing agent for nanoparticle stabilization, and its hydrophobic groups can protect the nanoparticles.^[24]^ Alternatively, it can adjust the polarity and increase the reaction efficiency through the formation of a short diffusion distance for oil‐ and water‐soluble substrates.^[25]^ A short diffusion distance was proposed by Yan et al. for the preparation of Janus mesosilica nanosheets with perpendicular mesochannels bearing palladium nanoparticles for the reduction of 4‐nitrophenol to 4‐aminophenol using NaBH_4_ as the reducing agent.^[25]^ They postulated that the assembly of Janus nanosheets at the readily accessible oil–water interface mixes the immiscible reaction compartments and leads to a 13‐fold improvement of the catalytic activity compared to a hydrophilic counterpart, and a 4.6‐fold improvement compared to a conventional silica‐based catalyst. The catalyst was recycled and reused five times with preservation of its activity. A Janus‐type filter material bearing silver nanoparticles was prepared by depositing Ag^+^ cations and a subsequent thermal treatment to stabilize the Ag nanoparticles on the surface.^[26]^ An interesting feature of this work is that at the end of the process, 4‐aminophenol was isolated with high efficiency from the permeable Janus filter by elution with ethyl acetate.

Janus dumbbell‐shaped mesoporous carbon‐PMO pairs (PMO=periodic mesoporous organosilica) were also found to be efficient catalysts for the reduction of nitroaromatic compounds.^[27]^ The material was prepared with a spherical shape on mesoporous resorcinol‐formaldehyde (RF) spheres by the hydrolysis of 1,4‐bis(triethoxysilyl)benzene (Figure 1b). The material was used for the reduction of some nitroaromatic compounds using NaBH_4_, even without stirring of the reaction mixture. For this purpose, platinum nanoparticles (1–5 nm in diameter) were selectively loaded onto the RF compartment and subsequently converted into mesoporous carbon spheres by carbonization under N_2_, thereby leading to a Pt/C&PMO Janus catalyst (Figure 1b). The material was able to form a Pickering emulsion in a water/toluene system with a droplet size of 30–120 μm (Figure 1c). The droplets remained stable for up to one month. The hydrogenation of some nitroaromatic compounds with electron‐donating and electron‐withdrawing groups, including 2‐nitrotoluene, 4‐nitrotoluene, 2‐nitroanisole, 4‐nitroanisole, and 1‐chloro‐4‐nitrobenzene, even in the absence of stirring, showed that the Janus catalyst had significant higher activities compared to a Pt/C (Pt loaded on carbon sphere) catalyst.

**Figure 2:**
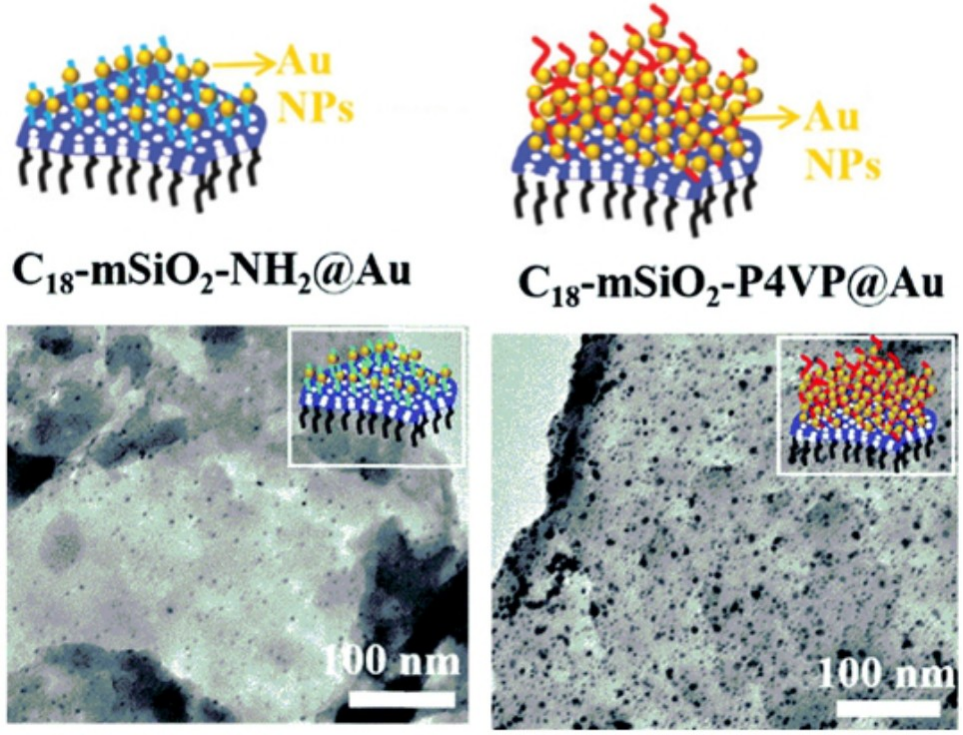
Schematic demonstration of the gold‐functionalized Janus catalysts and the corresponding TEM images, as reported by Lin et al. Reprinted and adapted from Ref. [28] with permission. © Royal Society of Chemistry (2020).

In 2020, Lin et al. reported amphiphilic Janus mesoporous nanosheets, which were selectively decorated on the hydrophilic surface with gold nanoparticles.^[28]^ The structure of the Janus catalysts as well as the corresponding TEM images are shown in Figure 2. They prepared two types of materials by employing ZIF‐67 as the core template for the particles.

The surface of the material was first coated with SiO_2_. This was followed by immobilization of either aminopropyl groups or bromine functionalities, which were obtained by reacting the surface amino groups with 2‐bromoisobutyryl bromide. Then, the ZIF‐67 template was removed by the addition of HCl. The controllable addition of poly(4‐vinylpyridine) (P4VP) by an atom transfer radical polymerization (ATRP) and of hydrophobic octadecyltrimethoxysilane (ODTMS) onto the surface of these two materials followed by the in situ growth of gold nanoparticles on the hydrophilic side of the nanosheets yielded two different types of Janus mesoporous nanosheets. TEM images revealed the uniform distribution of gold nanoparticles for both samples. However, the number of gold nanoparticles decorated on the C_18_‐mSiO_2_‐NH_2_@Au Janus nanosheets was clearly lower than that on the C_18_‐mSiO_2_‐P4VP@Au system (Figure 2), which was most likely due to the higher loading of coordinating groups in P4VP.

The material C_18_‐mSiO_2_‐P4VP@Au exhibited pH‐dependent catalytic properties for the reduction of 4‐nitrophenol as a result of the presence of P4VP on the surface. It has a higher activity compared to C_18_‐mSiO_2_‐NH_2_@Au Janus nanosheets at pH 6, while at pH 12, C_18_‐mSiO_2_‐NH_2_@Au showed the better catalytic efficiency. The lower activity of C_18_‐mSiO_2_‐P4VP@Au at the alkaline pH value was interpreted by the formation of hydrophobic aggregations of the initially hydrophilic P4VP chains ligated on the surface of the C_18_‐mSiO_2_‐P4VP@Au material. In addition, both materials showed good activity and recovery in a Pickering emulsion system comprising of water and decane as the solvents.

### 2.2 Oxidative Desulfurization and Dye Degradation

**Figure 3:**
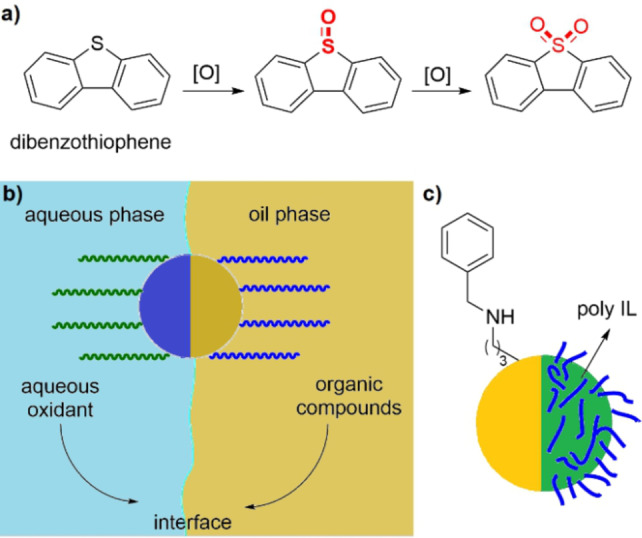
a) An example for the oxidative desulfurization of dibenzothiophene. b) Schematic demonstration of the action of an amphiphilic Janus catalyst for interfacial aqueous/organic reactions, in which the catalytic species can be incorporated in either the hydrophobic or the hydrophilic face of the material. c) The structure of the amphiphilic poly(ionic liquid)‐modified Fe_3_O_4_@SiO_2_ Janus catalyst.^[33]^

Oxidative desulfurization is an important reaction, with a particular emphasis for controlling environmental pollutants (Figure 3a). There is significant interest in using Janus catalysts in this context, since they can comprise a hydrophilic face for the diffusion of hydrogen peroxide (H_2_O_2_), which is often used as an aqueous oxidant and an organic face, which is compatible with the organic, sulfur‐containing compounds. As often, the interaction between the aqueous and the organic phase is a crucial factor for achieving high catalytic efficiency (Figure 3b). To address the application of a Janus catalyst for a desulfurization reaction, for example for fuel up‐grading, Janus nanosheets with hydrophobic phenyl groups on one side and silicotungstate‐, phosphotungstate‐, or phosphomolybdate‐containing ionic liquids on the other side were prepared by employing the oil in water method.^[29]^ The nanosheets exhibited good amphiphilic properties for the diffusion of aqueous H_2_O_2_ as well as sufficient hydrophobicity for dibenzothiophene—one of the major sulfur impurities in fuel. The material was used as both an emulsifier for the sulfur‐containing compounds and as an oxidation catalyst. The sulfur removal efficiency was improved from 68 % in a conventional biphasic system to 97 % in the presence of the Janus catalyst.

Janus materials were also employed for the catalytic reduction of dyes with NaBH_4_ ^[30]^ and their oxidative degradation, the latter being a more commonly used process. Following the concept shown in Figure 3b, Liang et al. designed some ionic liquid‐based systems for the oxidative degradation of dyes using aqueous H_2_O_2_ as the oxidant.^[31]^ A poly(ionic liquid)‐based Janus catalyst was prepared by immobilization of the PW_12_O_40_^3−^ anion onto one side of the material, which was decorated with hydrophobic silica‐phenyl groups on its other side.^[32]^ Water‐soluble methyl orange together with H_2_O_2_ can adsorb on the hydrophilic face of the material, while the hydrophobic degradation products are rapidly transferred into the organic phase consisting of toluene.

A poly(ionic liquid)‐modified Fe_3_O_4_@SiO_2_ Janus catalyst with phenyl groups and a poly(ionic liquid) on the two different sides was fabricated and used for the decomposition of methyl orange in an oil‐in‐water emulsion system.^[33]^ The hydrophobic face of this material was prepared by the reaction of the surface's aminopropyl groups with benzaldehyde in a Schiff‐base reaction and the subsequent reduction of the C=N double bond with NaBH_4_ (Figure 3c). The other face of the particle was selectively functionalized by “masking” one face of the particles in paraffin and modification of the other face with the ionic liquid 1‐vinyl‐3‐ethylimidazolium bromide. It was found that the degradation of methyl orange proceeded fast in the emulsion system and the catalyst could be recovered in a facile manner by using an external magnet. An interesting feature of this work was the adjustment of the stability of the emulsion by switching the anions of the Janus particles. The ionic liquid with Br^−^ or PW_12_O_40_^3−^ anions showed an amphiphilic character, whereas the Janus catalyst with a more hydrophobic ionic liquid containing PF_6_^−^ ions could only be dispersed in a hydrophobic phase (toluene).

### 2.3 Asymmetric Catalysis

The simple recovery and reusability of heterogeneous compared to homogeneous catalysts is highly beneficial, especially in asymmetric organic synthesis, as most chiral organic ligands or complexes are rather expensive.^[31]^ In addition, traces of metallic impurities originating from catalysts can lead to serious difficulties in the purification of pharmaceutical products.

**Figure 4:**
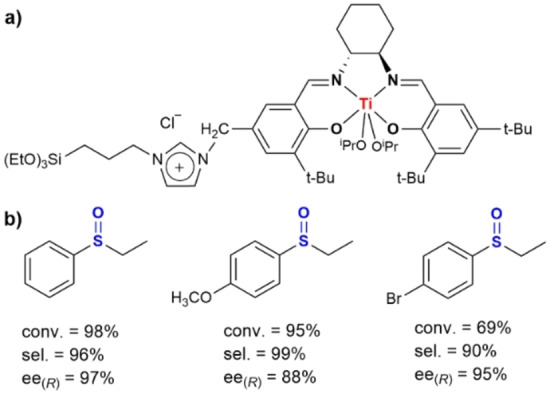
a) The molecular structure of the chiral Ti^IV^‐salen complex used by Tan et al.^[36]^ b) Some examples of sulfoxides derived from the aqueous‐phase oxidation of the corresponding sulfides.^[36a]^

Heterogeneous asymmetric catalytic reactions in water are a hot topic in organic synthesis, not only because water is an environmentally benign solvent, but also because it can play a positive role in improving the selectivity through the accumulation of organic substrates in the reaction mixture or in transiently formed droplets. However, a known problem of conventional heterogeneous catalysts is the poor mass transfer, which is why in some case the use of PTCs is necessary to improve the catalytic activity.^[35]^ Recently, Tan et al. employed the wax‐in‐water as well as the templated polystyrene method for the fabrication of Janus catalysts containing a chiral Ti^IV^‐salen species on one side and −OH groups on the other side of spherical particles.^[36]^ They used this catalyst for asymmetric sulfoxidation in water (Figure 4).^[36]^

The asymmetric sulfoxidation of various sulfides in water provided high yields and selectivities within 60 min at 25 °C with aqueous H_2_O_2_ as the oxidant (Figure 4b).^[36a]^ The major enantiomer had the *R* configuration. The authors found that the activity of the Janus ionic liquid based chiral Ti^IV^‐salen‐type catalyst was higher than a series of non‐Janus chiral analogues such as i) a heterogeneous catalyst with an unpolar spacer, ii) a silica catalyst with randomly distributed chiral ligands, and iii) the neat Ti^IV^‐salen complex. The high activity of the Janus catalyst was attributed to the formation of a Pickering emulsion during the reaction, in which water “pushes” the organic substrates towards the catalytically active chiral sites.

### 2.4 Biomass Transformation

**Figure 5:**
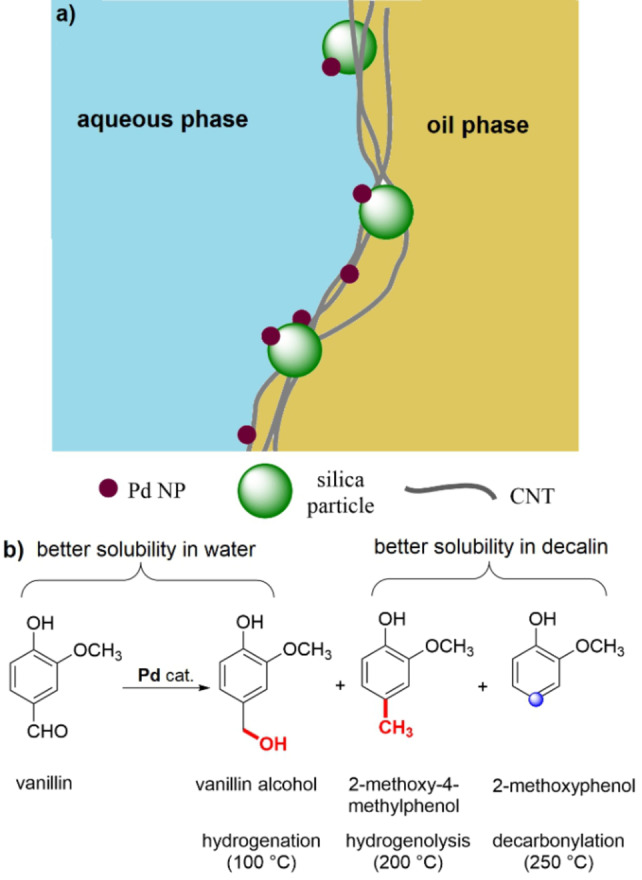
a) A graphical demonstration of interfacial palladium nanoparticles on Janus hybrid CNT/silica particles proposed by Crossley et al.^[37]^ b) The product distribution from the hydrodeoxygenation of vanillin as a function of the employed reaction temperature.

The production of high‐value chemicals from biomass is becoming more and more important in terms of a sustainable transformation in the chemical industry. In one of the first reports dealing with the application of a heterogeneous Janus catalyst, Crossley et al. described a hybrid system obtained by fusing carbon nanotubes (CNT) to silica particles followed by the addition of palladium nanoparticles (Pd NPs).^[37]^ In this interfacial catalyst, the hydrophilic oxides tend to attract water, while the carbon nanotubes turn towards the hydrophobic decalin phase. The addition of the palladium nanoparticles to the surface of the carbon nanotubes provides a bifunctional amphiphilic material, which can work efficiently in both phases (Figure 5a). The authors found that growing carbon nanotubes on the silica particles results in most of the palladium species binding to the surface of the silica particles. Hence, water‐soluble substrates such as vanillin can react more efficiently in such a system. The results of the hydrodeoxygenation of vanillin are shown in Figure 5b. The product distribution strongly depends on the reaction temperature. At 100 °C, the hydrogenation of vanillin leads to vanillin alcohol as the major product with a high partition in the aqueous phase (ca. 80 %). However, when the temperature is raised to 200 °C, 2‐methoxy‐4‐methylphenol (creosol) is formed, with 90 % in the decalin phase. 2‐Methoxyphenol could successfully be obtained by the decarbonylation of vanillin at 250 °C and with a more than 80 % distribution in the nonpolar phase. Such a biphasic synthesis is of interest, particularly when the target compound is soluble in the organic phase while undesirable side products remain in the aqueous phase.

**Figure 6:**
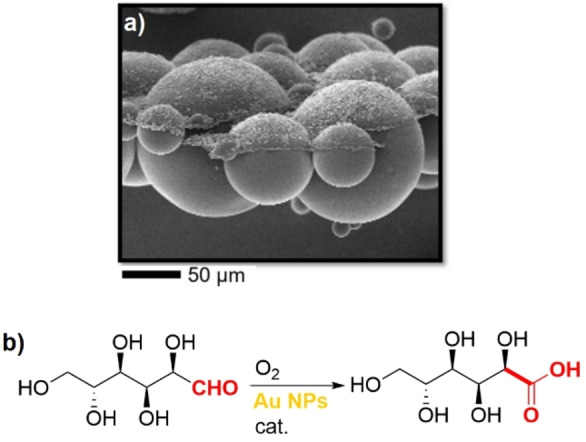
a) SEM image of the Janus particles after the treatment with ECA. Reprinted and adapted from Ref. [38]. © 2021 American Chemical Society under CC BY 4.0 license. b) Oxidation of D‐glucose to gluconic acid using gold (Au) NPs in the presence of perfluorinated Janus particles for facile O_2_ delivery (cat.**=**Janus particles with perfluoroalkyl/carboxylic acid functionalities).

The possible multifunctionalization of Janus particles provides the opportunity to prepare particles with specific organic groups that allow to improve the solubility of oxygen in the emulsion system. For example, Frank et al. prepared Janus particles with perfluoroalkyl/carboxylic acid functionalities.^[38]^ They showed that the addition of the perfluoroalkyl groups onto the surface of the polymer particles led to a higher solubility of oxygen. Furthermore, they employed an interesting technique to demonstrate the interfacial contact angles of the Janus particles at air–water interfaces (Figure 6a). For this purpose, the Janus particles were first deposited at an air–water interface and thereafter, these interfaces were solidified by a vapor treatment with an ethyl 2‐cyanoacrylate (ECA) based superglue. The result of this experiment showed that the determined interfacial contact angles were dependent on the Janus ratio of the particles and independent of their size distribution. The entrapped oxygen in the emulsion droplets increased the activity of the catalyst for the oxidation of D‐glucose to gluconic acid in the presence of gold nanoparticles by up to a factor of 2.2 after the addition of the Janus particles (Figure 6b). In this case, the higher specific surface area of the smaller sized particles had a higher impact on the increase of the reaction rate.

**Figure 7:**
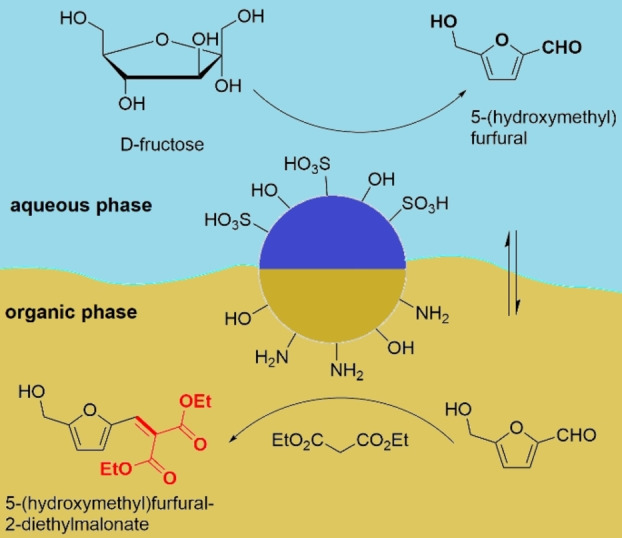
The structure of a bifunctional, acid‐base Janus catalyst employed for the tandem interfacial dehydration and subsequent Knoevenagel condensation of fructose in a Pickering emulsion.^[40]^

Employing bifunctional heterogeneous catalysts bearing incompatible groups (such as acids and bases) is an interesting strategy to conduct multistep organic reactions in one pot.^[39]^ Recently, Chang et al. prepared a bifunctional Janus catalyst with sulfonic acid (−SO_3_H) and amine (−NH_2_) groups for the interfacial dehydration of fructose followed by a Knoevenagel condensation in a Pickering emulsion.^[40]^ The acid‐base Janus particles were prepared by using monodisperse, hydrophobic, amine‐functionalized silica particles in an oil‐in‐water emulsion with a molten paraffin wax. The free hemisphere of the silica particles was selectively etched and then functionalized with the sulfonic acid groups. The structure of the catalyst and its application for the tandem dehydration‐Knoevenagel condensation process is shown in Figure 7.

To investigate the selective etching in more detail, the material was treated with silver nanoparticles. The authors found that the hemisphere with amine groups could be exclusively labeled, with the etched face remaining untouched. Additionally, it was found that the Janus silica particles were rapidly transferred to the interface between the water and toluene, which was interpreted as proof of the successful synthesis of an anisotropic structure. The loading of acid and amino groups on the material can be adjusted. The conversion of fructose into 5‐(hydroxymethyl)furfural increases with higher acid concentrations, while it decreases with a higher loading of amine groups. In contrast, the yield of the Knoevenagel condensation improves with a higher loading of amine groups.

### 2.5 Cascade Reactions

**Scheme 1:**
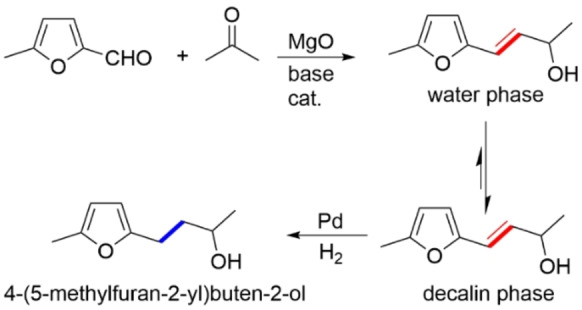
The interfacial cascade aldol condensation/reduction of 5‐methylfurfural to 4‐(5‐methylfuran‐2‐yl)buten‐2‐ol with a MgO/CNT/Pd Janus catalyst.^[37]^

The above‐mentioned example describing the dehydration of fructose and the subsequent Knoevenagel condensation of the resulting 5‐(hydroxymethyl)furfural is, viewed from a different angle, also an example of a cascade reaction. A cascade or tandem reaction (also known as a domino reaction) is a sequential chemical transformation involving at least two consecutive reactions in which the occurrence of the next reaction depends on the formation of the product in the previous step. For example, in the catalytic system described in Figure 5,^[37]^ the silica particles were replaced by magnesium oxide (MgO). Then, palladium nanoparticles were immobilized on the resulting organic/inorganic hybrid Janus catalyst. In the silica/CNT hybrid Janus material, the palladium nanoparticles were mainly bound to the surface of the silica particles (Figure 5). However, in the MgO/CNT hybrid material, the palladium catalyst was immobilized on both the CNT and MgO particles and was therefore able to catalyze a subsequent hydrogenation reaction in both the hydrophobic and hydrophilic phases of the emulsion. Magnesium oxide catalyzed the coupling reaction of 5‐methylfurfural and acetone, a process that was considered for biofuel production.^[37]^ The cascade aldol condensation/hydrogenation reaction of 5‐methylfurfural is shown in Scheme 1. An analysis of the phases at the end of the reaction showed that 16.4 and 2.5 weight % of the final product 4‐(5‐methylfuran‐2‐yl)buten‐2‐ol remained in the decalin and H_2_O phases, respectively.

**Figure 8:**
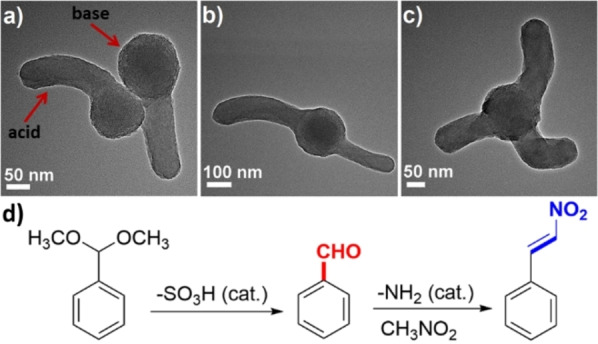
a)–c) Different morphologies of the Janus mesoporous hybrid silica with acid and base components. d) A cascade deacetalization/Henry reaction carried out with the Janus acid‐base catalyst. Reprinted and adapted from Ref. [41] with permission. © John Wiley and Sons (2015).

In a study reported by Wang et al., Janus mesoporous silica nanostructures with organic/inorganic hybrid components were fabricated using a sprout‐like growth method.^[41]^ Herein, mesoporous SiO_2_ branches grew on the surface of PMO nanoparticle seeds. The authors could successfully prepare different morphologies by varying the ratio of the PMO core and the SiO_2_ branches (Figure 8a–c).

The Janus nanocatalyst was modified with acidic −SO_3_H groups on the SiO_2_ branches and basic −NH_2_ functionalities on the PMO core. The catalytic performance of the material was tested in a cascade deacetalization/Henry reaction (Figure 8d). The results of the catalytic investigation showed that in the presence of the bifunctional acid‐base catalyst, almost all of the benzaldehyde dimethylacetal was converted into the target product. When a material containing only the acid groups was used, benzaldehyde was formed as the sole product, whereas when the monofunctional amine catalyst was used, no reaction took place since the acetal‐protecting group is stable in the presence of the basic catalyst.

**Figure 9:**
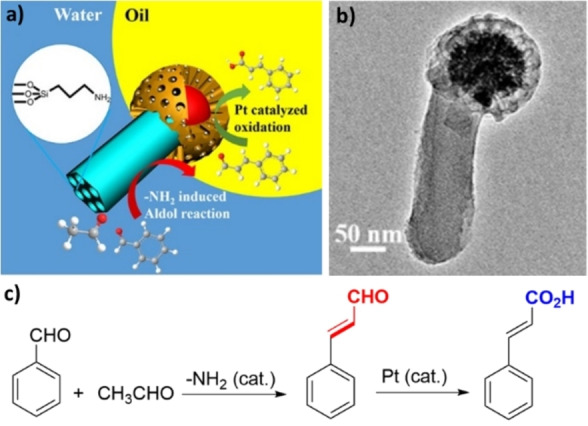
a) Schematic description of the Janus silica nanorods bearing platinum nanoparticles as catalysts on the hydrophobic carbon heads and b) the corresponding TEM image. Reprinted and adapted from Ref. [42] with permission. © American Chemical Society (2018). c) The cascade aldol condensation/oxidation of benzaldehyde.

A cascade aldol condensation/oxidation reaction in a Pickering emulsion system consisting of water and toluene was carried out with Janus silica nanorods bearing platinum (Pt) nanoparticles on the hydrophobic carbon heads (Figure 9).^[42]^ In this procedure, the water‐soluble reagents underwent aldol condensation in the presence of the basic −NH_2_ groups of the catalyst to form cinnamaldehyde, which subsequently diffused into the toluene phase, where it was oxidized to cinnamic acid in the presence of the platinum catalyst.

### 2.6 Oxidation

Oxidations are important transformations for the generation of synthetic building blocks and pharmaceuticals.^[43]^ H_2_O_2_ and oxygen (air) are broadly used as oxidants and have shown excellent compatibility with Janus‐type catalysts. An aqueous solution of H_2_O_2_ can, for example, be readily employed as a part of a Pickering emulsion, mediated by a Janus catalyst. Moreover, it was shown that the modification of Janus materials with perfluorocarbon groups can dramatically improve the solubility of oxygen.^[38]^

**Figure 10:**
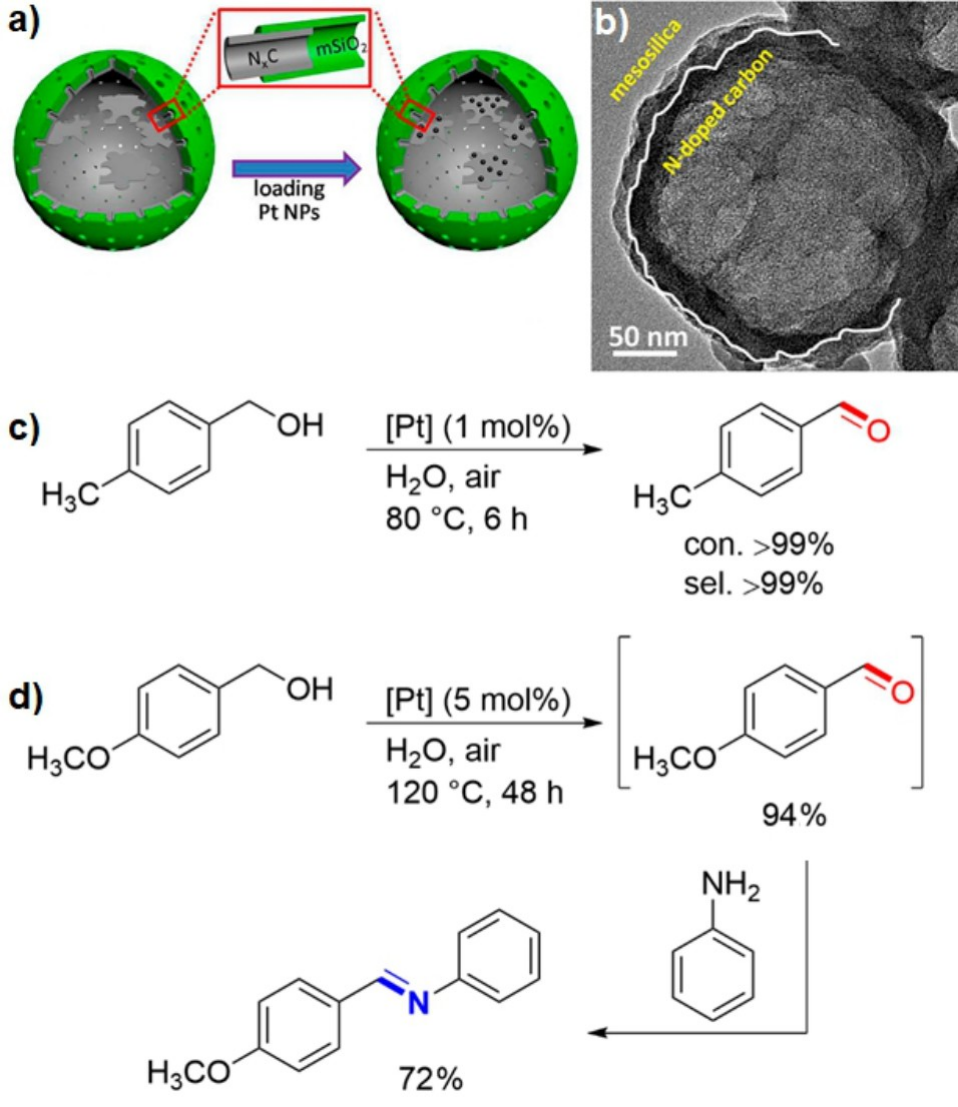
a) Platinum nanoparticles on the surface of Janus nitrogen‐doped carbon@silica hollow spheres and b) the corresponding TEM image. Reprinted and adapted from Ref. [44] with permission. © American Chemical Society (2018). c) Base‐free oxidation of alcohols and d) synthesis of imines from the reaction of aniline with the aldehyde intermediate.

A base‐free procedure for the selective aerobic oxidation of alcohols in water with Janus catalysts was developed by Dai et al. They used platinum nanoparticles on the surface of Janus nitrogen‐doped carbon@silica hollow spheres (Figure 10).^[44]^ Figures 10a and 10b show the schematic structure of the catalyst together with a TEM image. The material was prepared by selectively coating polybenzoxazine spheres with tetraethyl orthosilicate (TEOS) and the subsequent immobilization of ultrasmall platinum nanoparticles (<3 nm) to afford hollow spheres with an internal hydrophobic and an external hydrophilic surface. The TEM analysis proves the precise Janus geometry of the catalyst (Figure 10b). The role of the doped nitrogen atoms is to stabilize the platinum nanoparticles as well as to provide sufficient basicity, which is necessary for the reaction. In this procedure, various alcohols were efficiently and selectively converted into the corresponding aldehydes (Figure 10c). Furthermore, the catalyst was successfully used for the one‐pot synthesis of imines from different alcohols and amines in a tandem reaction (Figure 10d).

The selective oxidation of alcohols to aldehydes and ketones with H_2_O_2_ instead of oxygen was also investigated by designing a Pickering interfacial catalytic system containing a phosphotungstate‐functionalized mesoporous Janus material.^[45]^ This solid catalyst was prepared by protonation of surface‐bound aminopropyl groups with different amounts of phosphotungstic acid (H_3_PW_12_O_40_). The immobilized Janus catalyst showed higher activity and selectivity than H_3_PW_12_O_40_ in a homogeneous system.

**Figure 11:**
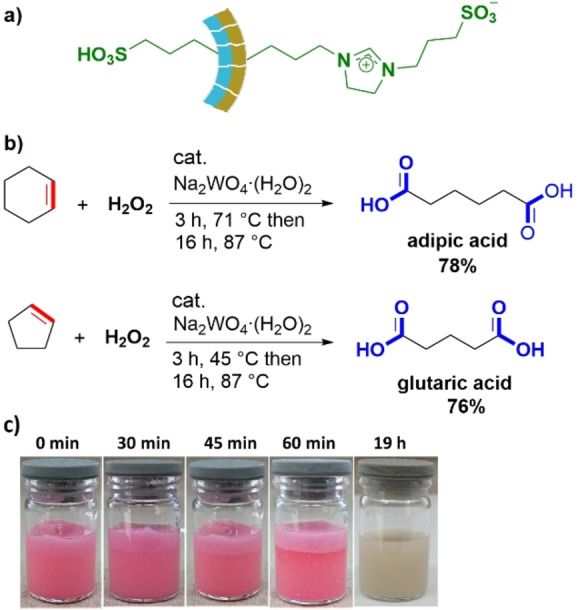
a) The structure of the surfactant‐like and water‐dispersible Brønsted acid catalyst used for aqueous‐phase oxidations. b) Oxidation of cyclohexene to adipic acid and cyclopentene to glutaric acid in the presence of the Janus catalyst. c) Images of the reaction mixture for the oxidation of cyclohexene at different times (0 to 60 min) in the presence of methyl orange solution as a pH indicator, and the image of the reaction mixture at the end of the reaction (without indicator, right). Reproduced and adapted from Ref. [42]. Copyright 2020 John Wiley and Sons under CC BY 4.0 license.

With the aim of finding a simple method with potential for the large‐scale synthesis of a Janus material, we recently disclosed a practical route by a modification of the so‐called Stöber process, which originally was developed for the generation of monodisperse silica particles.^[46]^ This procedure requires a basic water/ethanol/CTAB solution (CTAB=cetyltrimethylammonium bromide) containing *n*‐decane to assist the formation of hollow soft templates (micelles). The addition of TEOS results in the condensation of a mesoporous silica shell around these micelles. Starting from such materials, which have a hydrophobic inner core containing decane and an aqueous external environment, we prepared various Janus materials with diverse polarities. A highly water‐dispersible heterogeneous Brønsted acid surfactant was obtained by the selective immobilization of propyl−SO_3_H groups on one side and zwitterionic species on the other.^[46b]^ To achieve this, a hollow material with propyl−SH groups inside was prepared first, followed by the functionalization of the outer face with ionic moieties. The final catalyst was obtained after crushing the hollow spheres and subsequent oxidation of the −SH to −SO_3_H groups (Figure 11a). The polarity of the material was assessed by its perfect dispersion in water. This observation was further confirmed by a laser diffraction analysis of the particle size distribution, which indicated the presence of single particles without noticeable agglomeration.

**Figure 12:**
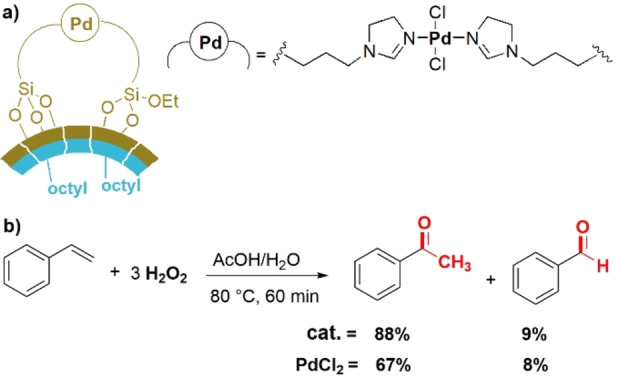
a) The structure of the single‐site palladium complex immobilized on the free face of the hydrophobic Janus nanosheets to form a Janus catalyst. b) The Wacker‐type oxidation of styrene to acetophenone in the presence of 1 mol % of the Janus catalyst and PdCl_2_.^[48]^

The activity of the catalyst was explored for the synthesis of carboxylic acid derivatives such as adipic and glutaric acids—which are important industrial building blocks—through the oxidative cleavage of cyclohexene and cyclopentene with H_2_O_2_ as the oxidant and catalytic amounts of Na_2_WO_4_⋅(H_2_O)_2_ (Figure 11b). Based on the results of the reactions and some mechanistic investigations, we conclud that the material acts as a heterogeneous surfactant, which can replace the traditionally used hazardous and expensive quaternary ammonium phase‐transfer catalysts. Figure 11c shows images of the initial reaction mixture and miscibility of the reactant as a function of time as well as the final reaction mixture for the oxidation of cyclohexene to adipic acid in the presence of a water‐dispersible Janus surfactant. There has been an increased demand to use aqueous H_2_O_2_ as a green oxidant during the last few years. The fact that water is the sole by‐product and that recent developments have made the production of H_2_O_2_ cheaper and safer are clear benefits of this strategy.^[47]^ A certain disadvantage related to oxidation reactions with H_2_O_2_ is its gradual decomposition, in particular at higher temperatures and concentrations. Therefore, the design of high‐performance catalytic systems that enable aqueous‐phase oxidations with short reaction times is an interesting strategy to reduce the decomposition rate of H_2_O_2_. In our recent study on the application of Janus catalysts, we synthesized a Janus material bearing a single‐site palladium complex on one face of hydrophobic silica nanosheets (Figure 12a).^[48]^ The activity of this catalyst was tested in a Wacker‐type oxidation of styrene with H_2_O_2_ to generate acetophenone (Figure 12b). The results indicated that acetophenone was obtained in 88 % yield after only 60 min at 80 °C by employing 1 mol % of the catalyst (based on palladium) in a mixture of acetic acid and water. Interestingly, we found that the activity of the catalyst was even higher than a comparable homogeneous PdCl_2_‐based system (Figure 12b). The activity of this catalytic system was explained by a highly efficient diffusion of the starting materials to the active sites. Styrene approaches from the hydrophobic face of the material and rapidly meets H_2_O_2_ and the palladium catalyst on a nanostructured interface provided by the Janus nanosheets.

### 2.7 Cross‐Coupling Reactions

Transition‐metal‐catalyzed cross‐coupling reactions have found widespread applications in (in)organic chemistry—ranging from the synthesis of simple industrial building blocks to valuable biologically active compounds.^[49]^ The immobilization of transition metals on the surface of Janus materials can not only improve the recovery and reusability of the catalyst, but the unique properties of Janus materials can also significantly improve the performance of cross‐coupling reactions.

**Figure 13:**
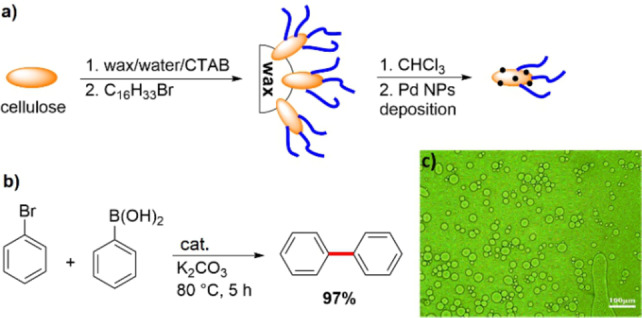
a) Immobilization of palladium nanoparticles on the surface of a modified Janus cellulose. b) The palladium‐catalyzed Suzuki coupling reaction of bromobenzene and phenylboronic acid in water using the Janus catalyst. c) The optical micrograph of the obtained emulsion for the Suzuki reaction in water. Reprinted and adapted from Ref. [50] with permission. © Royal Society of Chemistry (2020).

In 2020, a catalytic system based on palladium nanoparticles decorated on the surface of amphiphilic Janus‐type cellulose nanocrystals was prepared by Li et al. to perform interfacial Suzuki C−C coupling reactions in water.^[50]^ The material was prepared by the modification of trapped cellulose nanocrystals in wax with long‐chain organic groups and subsequent immobilization of palladium nanoparticles on the surface (Figure 13a).

The catalyst has two functions: it binds the palladium nanoparticles and acts as a phase‐transfer agent to conduct the reaction in water. The yield of the Suzuki reaction increased from 57 % with a comparable non‐Janus material to 97 % with the Janus palladium‐modified cellulose catalyst (Figure 13b). An optical micrograph image shows that the emulsion droplets are rapidly formed at the beginning of the reaction (Figure 13c).

**Figure 14:**
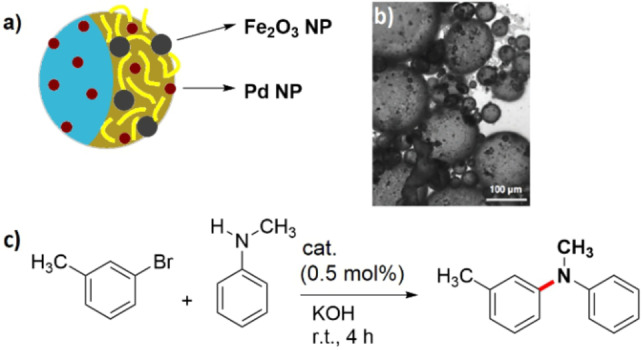
a) Schematic illustration of the magnetic responsive Janus microparticles bearing palladium nanoparticles. b) Bright‐field microscopy image of Pickering emulsions stabilized with Janus microparticles. Reprinted and adapted from Ref. [51] with permission. © Royal Society of Chemistry (2018). c) The Buchwald–Hartwig amination.

Amphiphilic and magnetic responsive Janus microparticles were found to be highly efficient catalysts for the Buchwald–Hartwig amination reaction in a Pickering emulsion (Figure 14a,b).^[51]^ The amphiphilic Janus microparticles were fabricated with two anisotropic hemispheres comprised of a hydrophilic and negatively charged poly(styrene‐*co*‐vinyl alcohol) (PS‐co‐VA) face and of a hydrophobic poly(tetradecyl acrylate) (PTA) face (Figure 14a). Dispersion of the particles in a solution of polyethyleneimine (PEI) led to the PS‐*co*‐PVA face becoming enriched with PEI groups. The addition of Fe_2_O_3_ and subsequently of palladium nanoparticles to these microparticles led to an immobilization of both types of nanoparticles at the PS‐*co*‐VA face and some palladium nanoparticles on the PTA face. The reported results for the Buchwald–Hartwig amination with this Janus catalyst (Figure 14c) indicated that the highest yields were obtained in a catalytic system comprising [(π‐allyl)PdCl]_2_ in combination with palladium nanoparticles on Janus microparticles (without Fe_2_O_3_). Although the reaction efficiency was reduced with the combination of [(π‐allyl)PdCl]_2_ and the Fe_2_O_3_‐containing support, the process still benefited from facile recovery by magnetic separation. The authors proposed that the Fe_2_O_3_ nanoparticles might also play a role in the reduction of palladium(II) to palladium(0).

**Figure 15:**
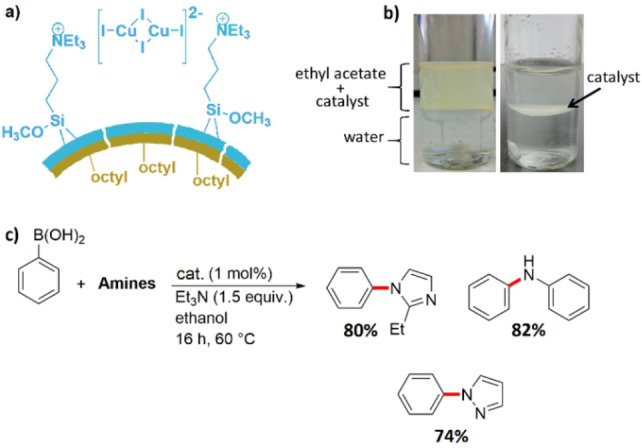
a) The structure of the heterogeneous dinuclear Janus copper catalyst. b) Initial suspension of the above Janus catalyst in a biphasic system of ethyl acetate and water (left) and migration of the catalyst particles towards the interface of the phases (right). Reprinted and adapted from Ref. [52] with permission. © American Chemical Society (2021). c) Some examples of amine derivatives prepared by the Chan–Lam reaction between phenylboronic acid and different amines.

The Chan–Lam reaction is an alternative to the palladium‐catalyzed C−N bond formation between a boronic acid and an amine as it allows the use of cheaper copper catalysts. However, to achieve high yields, large amounts of copper catalysts normally have to be applied.^[49c]^ After establishing the procedure for the synthesis of Janus catalysts by the modified Stöber process described above,^[46b]^ we prepared an ionic liquid based, heterogeneous Janus copper catalyst containing dinuclear Cu_2_I_4_^2−^ anions as the active species (Figure 15a).^[52]^ The catalyst is highly dispersible in organic solvents such as ethyl acetate or ethanol. However, in a biphasic water/ethyl acetate system, it migrates to the interface between the aqueous and organic phase and persists there for several days (Figure 15b). Deposition of the catalyst at the interface of two phases provides the opportunity for an in situ continuous extraction of the product(s) as a green technique for catalyst recycling. We found that the Chan–Lam reaction of a series of boronic acids and amines proceeds well when just 1 mol % of this catalyst is employed in ethanol (Figure 15c). The catalyst was recovered three times without substantial loss of activity. This finding is a significant improvement for the Chan–Lam reaction in terms of reaction costs and environmental issues.

### 2.8 Miscellaneous Organic Syntheses

The term “dehydration reactions in water” was introduced by Kobayashi et al. for some catalytic reactions in water which release water as a by‐product. In these reactions, surfactant‐like Brønsted acids are used as catalysts.^[53]^ They found that dodecylbenzenesulfonic acid (DBSA) is able to efficiently catalyze esterifications and some other relevant dehydration reactions through the formation of micelles in water. In the proposed mechanism, the authors describe that the hydrophobic tails of DBSA facilitate the water‐removal step and thereby shift the esterification equilibrium towards the ester products. On the basis of some similarities between Janus‐type materials and surfactants, acidic Janus materials can be considered as a heterogeneous variant of DBSA. Following this concept, several esterification reactions were developed using Janus‐type catalysts. For example, Cao et al. employed amphiphilic Janus nanoparticles with hydrophobic octadecyl and hydrophilic amine groups for the encapsulation of lipase.^[54]^ The agglomeration of nanosized Janus particles led to the formation of capsules with an average diameter of 5–50 μm. The authors showed that the activity of the encapsulated enzyme for the esterification of hexanoic acid with 1‐hexanol was 5.6 times higher than of the free enzyme in a biphasic system. Another process in the area of enzymatic esterification with Janus materials was established by the immobilization of lipase from *Candida rugosa* on the surface of amphiphilic Janus nitrogen‐doped carbon/MoS_2_ nanosheets. The catalyst was applied for the esterification of linolenic acid and phytosterol.^[55]^

**Figure 16:**
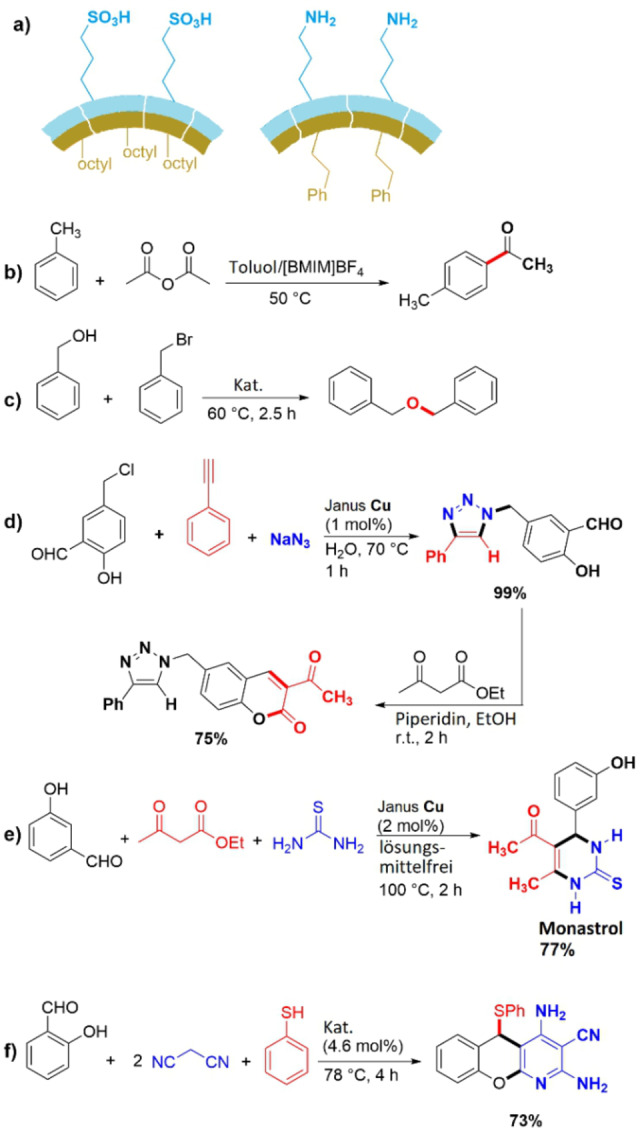
a) The structure of the Janus Brønsted acid and base organocatalysts.^[56]^ b) Friedel–Crafts acylation reaction using a Janus material functionalized with heteropoly acid in a non‐aqueous Pickering emulsion system consisting of toluene and [BMIM]BF_4_ (ionic liquid). c) Etherification reaction of benzyl alcohol with benzyl bromide catalyzed by Janus Fe_3_O_4_/polymer nanoparticles. d) Multicomponent reactions for the preparation of coumarin‐triazole derivatives using a Janus copper catalyst. e) Synthesis of monastrol through a solvent‐free Biginelli multicomponent reaction with a Janus copper catalyst. f) Four‐component synthesis of a functionalized benzopyranopyridine derivative using a basic Janus organocatalyst.

By mimicking a homogeneous Brønsted acidic surfactant, we prepared an amphiphilic heterogeneous catalyst with hydrophilic propylsulfonic acid and hydrophobic octyl groups on the surface (Figure 16a).^[56]^ The material was used for the esterification of ethanol and acetic acid with a nearly equimolar ratio of the reactants, which proceeded well even in the presence of up to 10 equivalents of added water. A laser diffraction analysis of the particle size distribution revealed that the particle size in the suspension ranged from 0.3 to 30 μm, which provided evidence for the formation of temporary micelle‐like agglomerations of the Janus nanosheets similar to those that were proposed when surfactant‐like DBSA was used in the homogeneous system.^[53]^

In a study reported by Xue et al., magnetic‐responsive Janus nanosheets with a phosphotungstate‐based ionic liquid were prepared and used for the esterification of oleic acid and methanol.^[57]^ The yield of the ester reached up to 80 % after 12 h at 70 °C. The catalyst was recycled four times, which is important since the material was prepared by a rather complicated reaction sequence involving several steps.

A heteropolyacid‐functionalized Janus material was also employed as an emulsifier and catalyst for the Friedel–Crafts acylation reaction between toluene and acetic anhydride in a continuous system (Figure 16b).^[58]^ This is an example of a non‐aqueous Pickering emulsion system using a combination of toluene and an ionic liquid ([BMIM]BF_4_). The results indicated that the catalyst could be recycled six times while preserving its high activity. The reaction was conducted in a flow reactor that was built with a permeable chromatography column. The mobile phase and the product could pass through the column, while the emulsion droplets and thus the real catalytic active species were retained in the column. Finally, the mixture was demulsified by centrifugation.

A magnetic quaternary ammonium Janus catalyst having Fe_3_O_4_ nanoparticles with high magnetic responsivity on one side and poly(GMA‐AA‐DVB) (GMA=glycidyl methacrylate, AA=acrylic acid, and DVB=divinyl benzene) on the other was employed as a heterogeneous PTC for the synthesis of dibenzyl ether from benzyl alcohol and benzyl bromide (Figure 16c).^[59]^ This composite material shows catalytic activity that is comparable to the molecular analogue tetrabutylammonium bromide (TBAB) but benefits from simple recycling and can be reused up to eight times. The key mechanistic feature of this Janus catalyst was ascribed to a perfect dispersion of the material in both the aqueous and the organic phase.

The performance of the Janus copper catalyst shown in Figure 15a was additionally studied in different multicomponent reactions for the preparation of some biologically active compounds, such as coumarin‐triazole derivatives (Figure 16d).^[52]^ Hereby, we first prepared a triazole derivative from a three‐component, copper‐catalyzed cycloaddition (CuAAC) between an alkyne, sodium azide, and 5‐(chloromethyl)‐2‐hydroxybenzaldehyde. Treatment of this compound with ethyl acetoacetate led to the formation of the coumarinylmethyl‐1,2,3‐triazole. Such structural motifs are considered as potentially antibacterial, anti‐inflammatory, and anticancer agents. Furthermore, this Janus catalyst was used for the synthesis of monastrol, an anticancer agent, through a Biginelli multicomponent reaction under solvent‐free conditions (Figure 16e).^[52]^

To extend the application of heterogeneous Janus organocatalysts, we also prepared a basic Janus organocatalyst (Figure 16a) and employed it for the Knoevenagel condensation of various aldehydes with malononitrile in water.^[56]^ This material could also be applied for the four‐component synthesis of a highly functionalized benzopyranopyridine derivative, another promising pharmaceutical scaffold (Figure 16f).

### 2.9 Electro‐ and Photocatalysis

Janus nanoarchitectures and nanoparticles have also found promising applications in electrocatalytic reactions, which are for several reasons related to green chemistry and renewable energy technologies.^[60]^ The improved electrocatalytic performance of such materials is mainly attributed to the increased mass diffusion and fluid slip, their high stability and structural strength, the increased number of active sites accompanied by a high accessibility to the active centers as well as a very low barrier for electron transfer arising from the anisotropic nature of the Janus nanoparticles.^[60]^

For example, a Janus electrocatalyst was prepared by sputtering titanium silicide (TiSi_*x*_) on one side of nitrogen‐doped carbon nanotubes followed by the deposition of platinum nanoparticles on the other side of the material.^[61]^ The activity of the catalyst was tested for the oxidation of methanol and was found to be higher than that of a commercial Pt/C catalyst in addition to a better CO tolerance (stability against poisoning).

**Figure 17:**
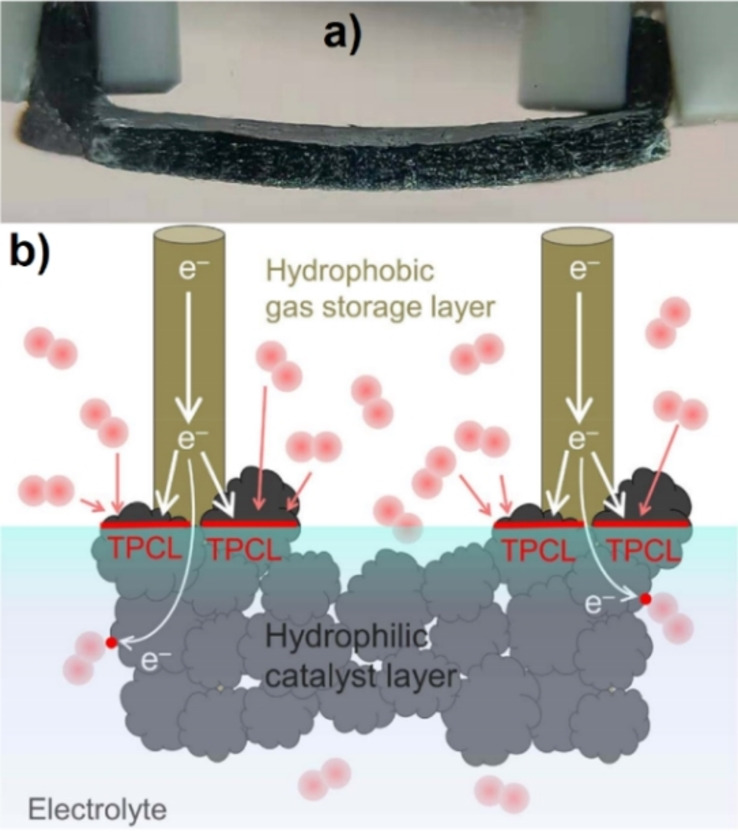
a) Side‐view picture of the Janus electrode. Reprinted and adapted from Ref. [62] with permission. © American Chemical Society (2020) b) Schematic illustration of the Janus electrode inside the electrolyte.

**Figure 18:**
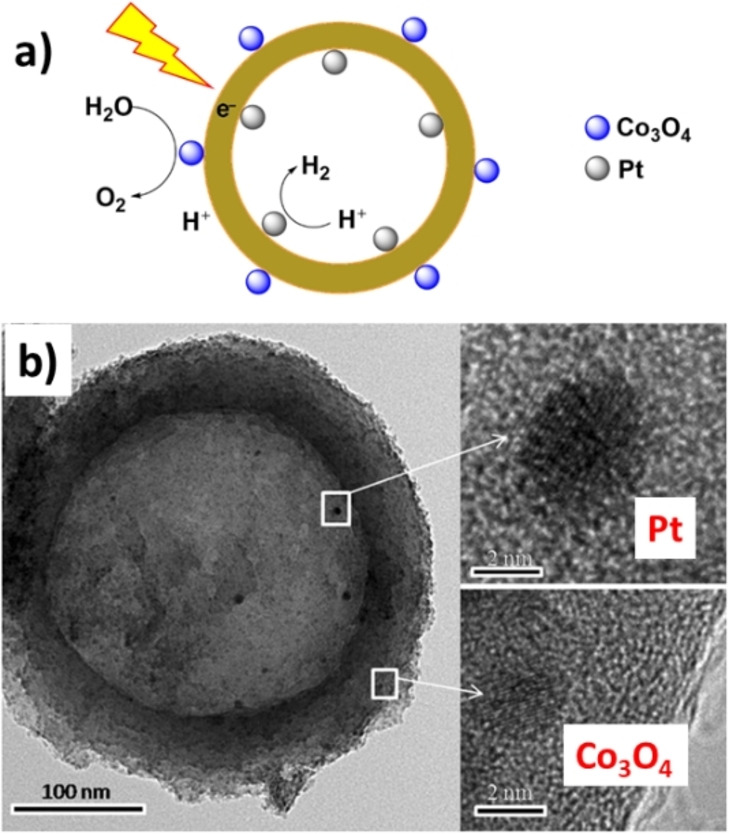
a) A schematic representation of the Janus platinum/Co_3_O_4_ nanoparticle catalyst used for the photocatalytic water splitting. b) TEM image of the Janus Pt/Co_3_O_4_ catalyst. Reprinted and adapted from Ref. [63] with permission. © John Wiley and Sons (2016).

In 2020, Zhang et al. prepared a Janus electrode for the production of H_2_O_2_ by an electrochemical oxygen reduction reaction.^[62]^ The material was fabricated with a hydrophobic graphite felt as the oxygen gas storage layer and oxidized carbon black/polyvinylidene as the hydrophilic layer of the catalyst. The key feature of this electrocatalytic system was the easing of the O_2_ mass transport limitation for the oxygen reduction reaction, which arises from the inherent low solubility and diffusivity of O_2_ in aqueous solutions, as well as the mitigation of the O_2_ bubble waste in the aeration process (circulation of air in a liquid) for the production of H_2_O_2_ (Figure 17). The authors explained that the contact between catalyst and electrolyte in the hydrophilic layer and the oxygen supply from the hydrophobic layer led to a much higher H_2_O_2_ concentration over the Janus electrode (62.1±2.6 mg L^−1^ at 30 min) compared to the solely hydrophobic (25.1±6.4 mg L^−1^) or hydrophilic (2.8±0.1 mg L^−1^) electrodes. The stability and durability of the catalyst was proved in ten continuous reaction cycles of 900 min, all showing a relatively constant efficiency. Therefore, the construction of such types of electrodes seems to be a notable strategy for electrocatalytic reactions in solid–liquid–gas intersections. Zheng et al. developed a photocatalytic water splitting Janus catalyst with platinum and Co_3_O_4_ nanoparticles on the inner and outer surfaces, respectively, of hollow carbon nitride spheres, with the aim to promote the surface redox properties (Figure 18).^[63]^ Assessment of the catalyst's activity revealed that the photocatalytic splitting of water (under a 300 W Xe lamp) led to the evolution of a stoichiometrically correct H_2_/O_2_ ratio of 2 : 1. The gas evolution rate of this Janus catalyst was about ten times higher than that of nonmetallic hollow carbon nitride spheres and three times higher than that of a material with a non‐Janus distribution of platinum and Co_3_O_4_ on the surface. A plausible explanation for the higher activity of the Janus catalyst lies in the spatially separated active sites for the evolution of H_2_ and O_2_, which prevents the regeneration of water by the reverse reaction and the mitigation of the charge recombination as a result of the unidirectional migration of the electrons and holes on the Janus surface.

Janus materials have been widely employed for photocatalytic reactions, although in some cases they did not possess a perfectly anisotropic topological structure.^[64]^ Some interesting examples refer to the preparation of Janus palladium‐gold particles for the photocatalytic reduction of CO_2_ to CH_4_,^[65]^ gold/CdSe particles for the photocatalytic generation of hydrogen,^[66]^ gold/SnS_2_ nanoparticles for the degradation of methyl orange,^[67]^ [TiO_2_/C]//[Bi_2_WO_6_/C] particles for the evolution of hydrogen and the degradation of organic pollutants,^[68]^ and magnetic Janus nanocomposites with iridium(III) complexes for a photoinduced electron/energy‐transfer reversible‐addition fragmentation chain‐transfer (PET‐RAFT) polymerization.^[69]^

The photodegradation of water‐soluble dyes in a Pickering emulsion system is an alternative for oxidative degradations (see Section 1.2). It works without the need for an external oxidant. For example, methyl‐capped Janus TiO_2_−SiO_2_ particles were synthesized and used as an emulsifier and a photocatalyst for the degradation of water‐soluble dyes such as methylene blue upon exposure to a 300 W ultraviolet (UV) lamp (*λ*=254 nm).^[70]^ The material is composed of silica rods grown on photoactive anatase TiO_2_ spheres, whose surface was further modified by methyl groups. Dispersion of the Janus nanocatalyst in a biphasic system of water and octane led to the formation of a highly stable and tunable emulsion (water‐in‐oil or oil‐in‐water), which provided a medium for the complete degradation of methylene blue even under static conditions (50 min under stirring versus 180 min under static conditions).

### 2.10 Gas‐Phase Reactions

The unique anisotropic structure of Janus materials was also found to have a synergistic effect for catalytic gas–phase reactions. Very recently, Deng et al. employed Au−CuO Janus nanoparticles with an average particle size of 3.8 nm for the oxidation of 2‐propanol to acetone in the gas phase. This reaction has a high potential for industrial applications.^[71]^ They found that at 100 °C and under atmospheric pressure, more than 97 % of 2‐propanol could be converted into acetone with a very high selectivity of more than 99 %. The high efficiency of the Janus gold‐CuO particles compared to similar non‐Janus gold or CuO catalysts is attributed to a better β‐C−H bond activation of 2‐propanol, a more efficient adsorption of 2‐propanol, and a noticeable decrease of the β‐C−H bond scission energy barrier, which was verified by DFT calculations.

**Figure 19:**
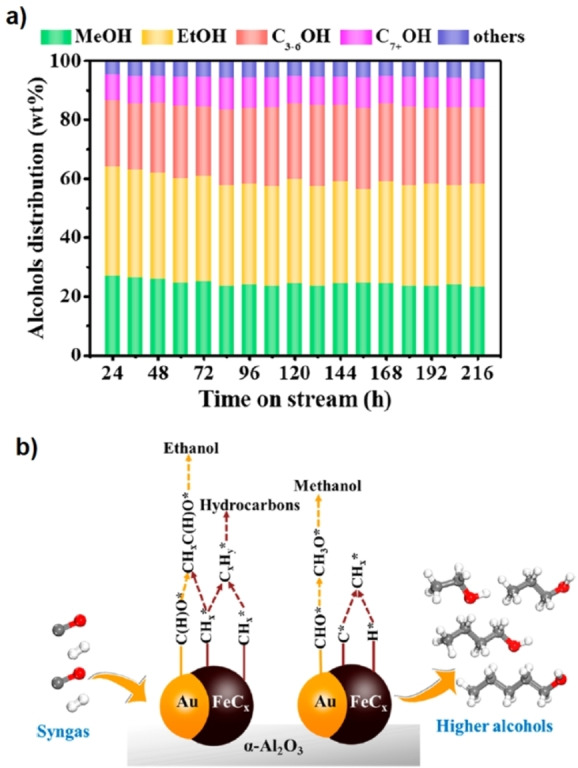
a) Distribution of alcohols derived from the catalytic conversion of syngas over Janus gold/iron catalyst. b) The proposed reaction mechanism for the synthesis of alcohols from syngas over the Janus catalyst. Reprinted and adapted from Ref. [72] with permission. © American Chemical Society (2021).

Syngas is a well‐known mixture composed of CO and H_2_ with widespread applications in the chemical industry. A Janus catalyst with dual gold‐Fe_2.2_C metallic sites was employed for the direct conversion of syngas into higher alcohols.^[72]^ In a study reported by Zeng et al.,^[72]^ the authors prepared an iron‐gold Janus catalyst and found that nanoparticles with the best Janus geometry were achieved with a Fe/Au molar ratio of 11.6. The Janus catalyst was prepared by the decomposition of iron oleate in the presence of gold seeds followed by the immobilization of the resulting gold‐Fe_3_O_4_ NPs on the surface of α‐Al_2_O_3_. The final active gold sites and iron carbide species are prepared by calcination, reduction, and subsequent pyrolysis of this material. The catalyst was employed for the synthesis of alcohols from syngas and shows higher activity compared to non‐Janus materials with the same composition. The performance of the catalyst for the production of higher alcohols is preserved for up to 216 h without a noticeable drop in activity. The product distribution as a function of time is shown in Figure 19a. According to the proposed mechanism (Figure 19b), the anisotropic surface of the catalyst is a key factor to enhance both insertion and dissociation of CO gas. In brief, gold sites are mainly responsible for the nondissociative CO adsorption and insertion, while FeC_*x*_ sites are mainly responsible for the dissociative adsorption and hydrogenation of CO to form CH_x_* species. The resulting CH_x_* species on the FeC_*x*_ centers can couple with each other, which finally leads to the formation of the long‐chain alcohols.

## 3 Summary and Outlook

In this Minireview, we summarize the latest and breakthrough catalytic features of task‐specific Janus materials in chemical reactions. The rational design of Janus catalysts with chemically distinct compositions and tunable polarities and functionalities offers the possibility to conduct chemical reactions in organic solvents, in water, or in biphasic water/organic mixtures (Pickering emulsion). Janus catalysts with polar groups such as ionic liquid moieties are, in particular, suitable for organic reactions in water, for the employment of aqueous hydrogen peroxide, and for reactions that include water‐soluble reducing agents. In contrast, it has frequently been shown that a lipophilic catalyst surface is in general suitable for water‐sensitive substrates having more organic characteristics. The most interesting type of Janus catalysts are amphiphilic materials having the capability to form a Pickering emulsion. Organic syntheses in Pickering emulsion systems have been widely developed and benefit from the excellent control and simple modification of the particle's wettability. In contrast to expensive and environmentally suspect traditional molecular surfactants, heterogeneous Janus catalysts can be easily separated from the reaction mixture. Maybe the most important reason for the higher efficiency of the above‐mentioned catalysts is the improved mass transport, which has also been observed for electro‐ and photocatalytic reactions.

During the last few years, in particular, it was shown that it was possible to use and modify different established synthetic procedures for the preparation of Janus materials.^[18]^ Although some nanoparticles may show dramatic changes in their catalytic activity, it has been shown that catalytic systems based on single‐chain Janus nanoparticles demonstrate enhanced activity at an emulsion interface.^[73]^ Although the large‐scale synthesis of the Janus materials is relatively unproblematic,^[74]^ some challenges remain for further catalytic applications. A notable example is the preparation of heterogeneous catalysts based on Janus bifunctional PMOs. Recently, we reported the one‐pot synthesis of a Janus bifunctional PMO with a highly anisotropic structure and the ability to form a stable a Pickering emulsion.^[75]^

Another challenge is the lack of a general procedure for the synthesis of Janus materials with “absolute selectivity” while keeping a precise surface functionality. Some procedures suffer from multiple steps (that may reduce the final yield of the material and increase costs), some time‐consuming procedures, and the employment of polymeric templates, which in industrial‐scale synthesis are not cost‐effective. At present, the preparation of Janus materials with incompatible moieties such as acid‐base or redox pairs is still not an easy task to do.

Single‐^[76]^ and dual‐atom^[77]^ heterogeneous catalysts are an innovative field of research that produces high‐performance metal catalysts with high catalytic efficiency. In such an atom‐scale catalysis, the choice of the host is crucial, since it influences both the electronic and steric properties of the metal centers. To the best of our knowledge, there is no explicit procedure for the preparation of “Janus single (or dual) atom heterogeneous catalysts” or “single (or dual) atom catalysts based on Janus nanoarchitectures”. In the case of the successful development of such heterogeneous catalysts, another door entitled “single (or dual) atom catalysis in a Pickering emulsion system” will be opened. The development of such heterogeneous catalytic systems can only be achieved once the structure–reactivity relationships that influence the synthesis are better understood. This is something to look forward to. Furthermore, the same logic can be extended to Janus metal organic frameworks (Janus‐MOFs)^[78]^ and Janus covalent organic frameworks (Janus‐COFs),^[79]^ which are still underdeveloped.

Computational simulation is an important tool to reveal various unknown features of Janus particles, such as self‐assembly in solution or in Pickering emulsions, prediction of aggregation and adsorption behavior at interfaces, aggregation kinetics, and colloidal stability.^[80]^ Finally, when designing new task‐specific heterogeneous Janus catalysts, the biocompatibility, biodegradability, and toxicity of the synthetic procedures as well as of the resulting materials need to be considered.

We hope that this Minireview will provide some detailed insights into the recent development and preparation of more efficient catalytic systems based on task‐specific Janus materials and will capture the imagination of researchers.

## Conflict of interest

The authors declare no conflict of interest.

## Biographical Information

*Majid Vafaeezadeh received his PhD in Chemistry at Sharif University of Technology, Tehran, Iran. In 2018, he joined in the group of Prof. Werner R. Thiel at TU Kaiserslautern as a postdoctoral researcher, financially supported by a Georg Forster Research Fellowship from the Alexander von Humboldt foundation. Currently, he is working as a scientific researcher in this research group. His research interests focus on the synthesis and applications of Janus nanomaterials for heterogeneous catalysis*.

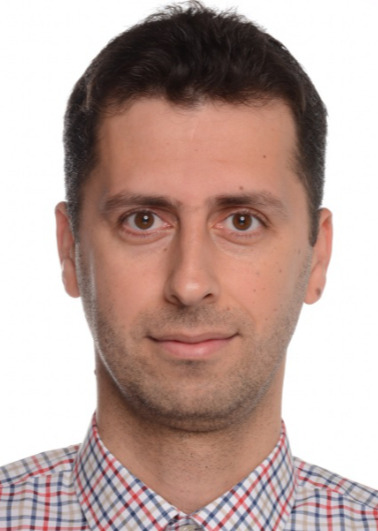

## Biographical Information

*Werner R. Thiel received his PhD in 1990 in Chemistry at TU München with Wolfgang A. Herrmann. After a postdoctoral fellowship in the group of Didier Astruc at Université de Bordeaux I, he started his academic career back in Munich. In 1997 he habilitated, in 2000 he was appointed associate professor at TU Chemnitz, and in 2004 he became full professor at TU Kaiserslautern. Since 2020 he has been vice‐president for research and technology at this university. His research focuses on fundamental aspects of homogeneous and single‐site heterogeneous catalysis*.